# Imprinting methylation errors in ART

**DOI:** 10.1007/s12522-014-0183-3

**Published:** 2014-06-19

**Authors:** Hitoshi Hiura, Hiroaki Okae, Hatsune Chiba, Naoko Miyauchi, Fumi Sato, Akiko Sato, Takahiro Arima

**Affiliations:** ^1^ Department of Informative Genetics, Environment and Genome Research Center Tohoku University Graduate School of Medicine 2‐1 Seiryo‐cho, Aoba‐ku 980‐8575 Sendai Japan

**Keywords:** Assisted reproductive technologies (ART), DNA methylation, Genomic imprinting, Intracytoplasmic sperm injection (ICSI), In vitro fertilization (IVF)

## Abstract

There has been an increase in incidence reports of rare imprinting disorders associated with assisted reproductive technology (ART). ART, including in vitro fertilization and intracytoplasmic sperm injections, is an important treatment for infertile people of reproductive age and increasingly produces children. The identification of epigenetic changes at imprinted loci in ART infants has led to the suggestion that ART techniques themselves may predispose embryos to acquire imprinting errors and diseases. In this review, we note that the particular steps of ART may be prone to induction of imprinting methylation errors during gametogenesis, fertilization and early embryonic development. In addition, we explain imprint‐associated diseases and their causes. Moreover, from a Japanese nationwide epidemiological study of imprint‐associated diseases, we determine their associations with ART. Epigenetic studies will be required to understand the pathogenesis, ART‐related risk factor(s) and what precautions can be taken to prevent the occurrence of input methylation errors. We hope that the constitution of children born after each ART procedure will reveal the safest and most ethical approach to use, which will be invaluable for the future development of standard ART.

## Introduction

Numerous studies published over the last few years have suggested that there is an increased incidence of rare imprint‐associated disorders associated with human assisted reproductive technologies (ART) [1, 2, 3, 4, 5, 6, 7, 8, 9] (Table 1). ART are important treatments for infertile people of reproductive age in which the eggs and/or sperm are manipulated in the laboratory. In Japan, 27,682 children were born after nearly 250,000 ART procedures [mainly in vitro fertilization (IVF) and intracytoplasmic sperm injection (ICSI)] in 2010 (Japan Society of Obstetrics and Gynecology). ART involve the isolation, handling, and culture of gametes and early embryos and ovarian stimulation at times when the epigenetic marks at imprinted loci are potentially vulnerable to external environmental influences. These techniques are associated with an increased risk of imprinting disorders, including cases of BWS (Beckwith–Wiedemann syndrome; NIM130650) and AS (Angelman syndrome; NIM105830) [5, 6, 7, 8]. Both IVF and ICSI are associated with the increased risk of imprinting disorders, though it is not clear at what point these imprinting errors arise [10, 11].

**Table 1:** ART and imprinting diseases

Disease	Treatment	Total	Samples	Observations	Reference
BWS	IVF/ICSI		7	*LIT1* LOM (5/6), *H19* GOM (1/6)	Debaun et al. [5]
	IVF (4)/ICSI (2)	149	6	*LIT1* LOM (6/6)	Gicquel et al. [6]
	IVF (3)/ICSI (3)	149	6	*LIT1* LOM (2/2)	Maher et al. [7, 31]
	IVF (3)/ICSI (1)	37	4	*LIT1* LOM (3/3)	Halliday et al. [9]
	IVF (12)/ICSI (5)	341	19	–	Chang et al. [40]
	IVF (8)/ICSI (3)	40	11	*LIT1* LOM (11/11), *IGF2R* LOM (2/11), *MEST* LOM (0/11), *SNRPN* LOM (1/11)	Rossignol et al. [39]
	IVF (1)/ICSI (5)	79	11	*LIT1* LOM (4/4)	Sutcliffe et al. [70]
	IVF (4)		6	*LIT1* LOM (4/4)	Doornbos et al. [11]
	IVF (12)/ICSI (13)		25	*LIT1* LOM (24/25), *MEST* LOM (2/25), *SNRPN* LOM (1/25), *PLAGL1* LOM (1/25)	Lim et al. [37]
	ICSI (1)		7	*ZDBF2* GOM (1/1), *MEST* GOM (1/1), *LIT1* LOM (1/1), *GNAS*‐*AS1* LOM (1/1)	Hiura et al. [32]
AS	ICSI (2)		2	*SNRPN* LOM (2/2)	Cox et al. [1]
	ICSI (1)		1	*SNRPN* LOM (1/1)	Orstavik et al. [8]
	ICSI (3)	79	3	*SNRPN* LOM (1/3), maternal deletion 15q11 (2/3)	Ludwig et al. [71]
SRS	ICSI (2)		2	–	Svensson et al. [72]
	IVF (1)		1	–	Galli‐Tsinopoulou et al. [73]
	IVF (1)		1	*MEST* GOM (1/1)	Kagami et al. [54]
	IVF (1)		1	–	Kallen et al. [74]
	ICSI (5)		15	*H19* LOM (5/5), *GRB10* GOM (2/5), *PEG10* GOM (1/5), *MEST* GOM (1/5), *ZNF597* LOM (1/5)	Hiura et al. [32]
RB	IVF (5)		5	–	Moll et al. [33]

Genomic imprinting confers different functions on the two parental genomes during development by silencing one allele of each imprinted gene in a parent‐of‐origin‐dependent manner [12, 13, 14, 15]. Imprinting accounts for the requirement of both maternal and paternal genomes in normal development and plays significant roles in regulating embryonic growth, placental function and neurobehavioral processes [16, 17]. Aberrant expression of some imprinted genes has been linked to a number of human diseases, developmental abnormalities and malignant tumors [18]. The epigenetic modifications that are imposed during gametogenesis act as primary imprint markers to distinguish the maternal and paternal alleles [14]. The most likely candidate for the gametic mark is DNA methylation. Allele‐specific DNA methylation has been observed in the vicinity of most imprinted genes. In some instances, the methylation is present on the inactive gene, suggesting a role for DNA methylation in silencing of the gene. DNA methylation is both a heritable and reversible epigenetic modification that is stably propagated after DNA replication. To transmit this epigenetic mark from one generation to the next, the imprints have to be erased in primordial germ cells (PGCs) [19, 20] and reestablished during gametogenesis in a sex‐specific manner.

The risks of ART cannot easily be evaluated because patients who receive ART may differ both demographically and genetically from the general population. Usually, patients requesting ART have lower fertility rates, increased reproductive loss rates and are of advanced age, all of which are associated with various fetal and neonatal abnormalities. All these confounding factors make it difficult to evaluate and estimate the risk. It is also difficult to determine the role of imprinting errors in any abnormality in patients conceived after ART. In this review, we will introduce the association between ART and imprinting‐related diseases in Japan and compare the molecular mechanisms of infants born after the use of ART and natural conception, which might provide clues to what leads to imprint‐associated disorders and identify ART‐related risk factors.

## Genomic imprinting and DNA methylation

**Figure 1:**
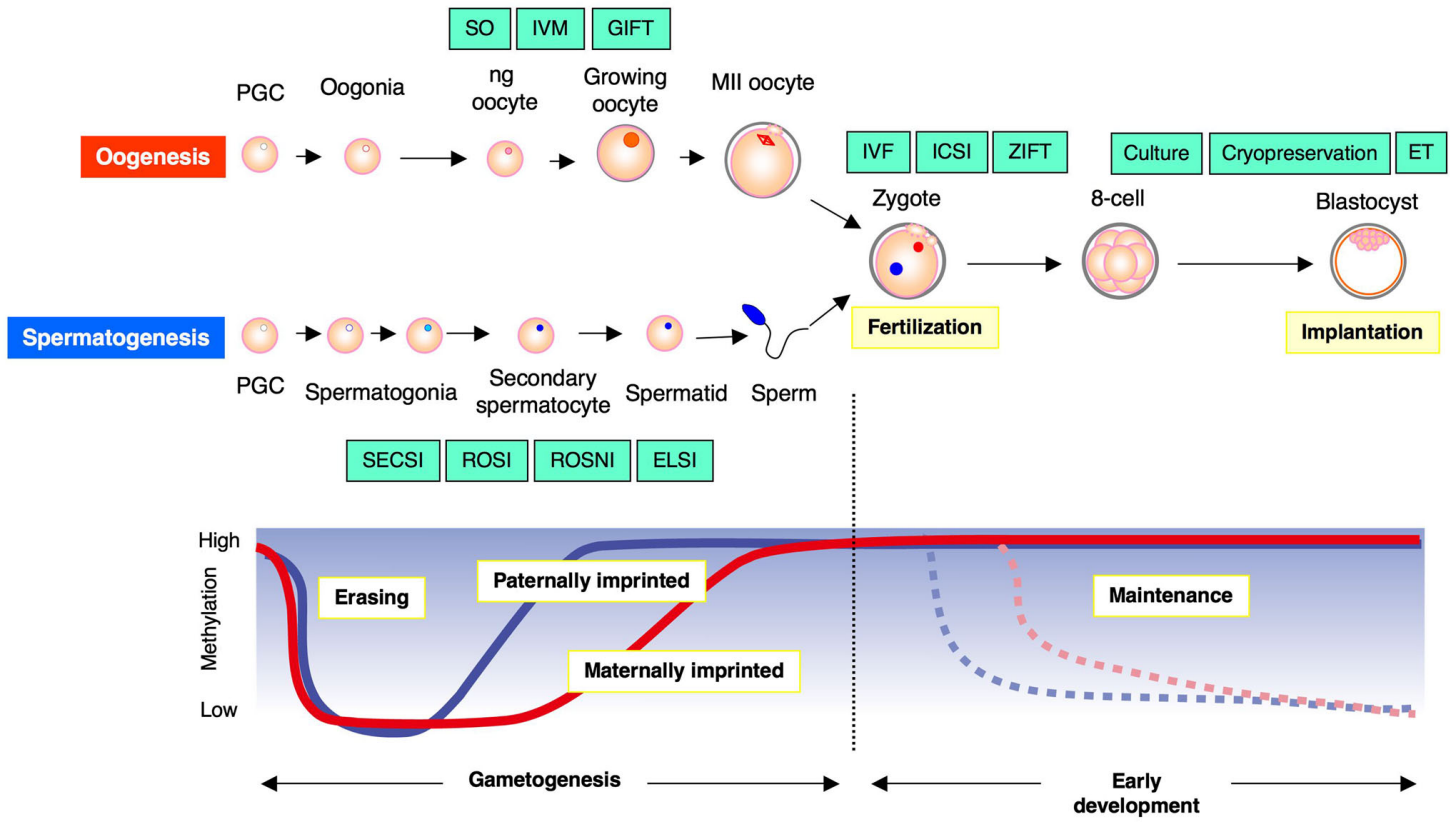
Methylation imprints in gametogenesis and the ART procedure. Genomic imprinting is a gamete‐specific modification (DNA methylation) that causes differential expression of the two parental alleles. During the transition from primordial to antral follicles in the postnatal growth phase (post‐pachytene), methylation is acquired asynchronously in a gene‐specific manner in mouse oogenesis. In sperm, imprint methylation is initiated prenatally before meiosis and is completed by the pachytene phase of postnatal spermatogenesis. The imprints of gametes are maintained stably in the early embryo despite overall epigenetic reprogramming. *IVM* In vitro oocyte maturation, *SO* superovulation, *GIFT* gamete intrafallopian transfer, *ZIFT* zygote intrafallopian transfer, *IVF* in vitro fertilization, *ICSI* intracytoplasmic sperm injection, *SECSI* secondary spermatocyte injection, *ROSI* round spermatid injection, *ROSNI* round spermatid nucleus injection, *PGC* primordial germ cell

Genomic imprinting, the allele‐specific expression of certain genes, accounts for the requirement for both maternal and paternal genomes in normal development and plays important roles in regulating embryonic growth, placental function and neurobehavioral processes [14, 15]. Many imprinted genes have been found to make clusters in some chromosomal regions. Their monoallelic expression relies on epigenetic mechanisms. DNA methylation of CpG‐dinucleotides at differentially methylated regions (DMRs) is an epigenetic mark (imprint methylation) and acts as an imprint control center (ICR). Imprint methylation resetting involves erasure of imprints in primordial germ cells (PGCs) and the acquisition of new sex‐specific imprints. Oocytes are arrested at prophase I, and, during the transition from primordial to antral follicles in the postnatal growth phase (post‐pachytene), methylation is acquired asynchronously in a gene‐specific manner in mouse oogenesis [21, 22, 23] (Fig. 1). In the human oocyte, a few reports have shown that the maternal methylation of these genes has already been initiated to some extent in adult non‐growing oocytes but not in neonatal oocytes [24]. In male sperm, imprint methylation (*H19*, *Rasgrf1* and *Gtl2*) is initiated prenatally before meiosis and completed by the pachytene phase of postnatal spermatogenesis [25, 26, 27, 28] (Fig. 1). Importantly, DNA methylation of genomic imprinting is established before fertilization during gametogenesis. The imprints of gametes are maintained stably in the early embryo despite overall epigenetic reprogramming [29]. The aberrant expression of several imprinted genes has been linked to a number of congenital diseases and malignant tumors in humans [18].

## Imprint‐associated disorders

**Figure 2:**
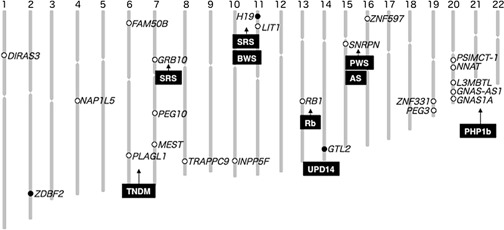
Methylation imprint chromosomal map in human and imprinted disorders. Twenty‐three human DMRs, 3 paternal (*black*) and 20 maternal DMRs (*white*) are confirmed. *BWS* Beckwith–Wiedemann syndrome, *AS* Angelman syndrome, *PWS* Prader–Willi syndrome, *SRS* Silver–Russell syndrome, *TNDM* transient neonatal diabetes mellitus, *RB* Retinoblastoma, *UPD14* uniparental disomy 14, *PHP1b* pseudohypoparathyroidism type 1b

Congenital imprinting disorders [BWS, AS, PWS (Prader–Willi syndrome; NIM176270) and SRS (Silver–Russell syndrome; NIM180860)] are rare diseases. It is known that they are caused by uniparental disomy (UPD), duplications, gene mutation (deletion), and aberrant DNA methylation in a specific region (Fig. 2). However, there are still many unidentified cases. Table 2 shows the characterization of these diseases.

**Table 2:** Characterization of congenital imprinting diseases

Disease	BWS	AS	PWS	SRS
Symptom	Exomphalos, macroglossia, gigantism. In 10 % of the cases, patients develop embryonal tumors	Global developmental delay, convulsions, scoliosis, excessive laughter, movement and balance disorder, sleep disorder, etc.	Hypothalamic dysfunction, disorder of satiety center, thermoregulatory center and respiratory center occurs	Severe intrauterine growth retardation, poor postnatal growth, craniofacial features such as a triangular shaped face and a broad forehead, body asymmetry, a variety of minor malformations
Disease	TNDM	RB	PHP1b	UPD14
Symptom	Patients are born with intrauterine growth retardation and present within the first 6 weeks of life with severe failure to thrive, hyperglycemia and dehydration	This disorder is noticed most commonly because of leukocoria. Besides, crossed eyes, deterioration of vision, corneal opacity, conjunctival injection and mydriasis are also typical symptoms	Renal PTH resistance is the primary defect, which causes hypocalcemia and hyperphosphatemia	Maternal UPD14 are IUGR, short stature, scoliosis, hypotonia, obesity, developmental delay, precocious puberty, a distinctive facial appearance and truncal obesity onset at 2–3 years. Paternal UPD14 is less common and phenotype is more severe

The cause of both PWS and AS is present on chromosome 15q11–13, but their phenotypes are entirely different. PWS is mainly caused by UPD (70 %) and methylation defects (2–5 %) of the paternal allele. PWS presents with endocrine and neural defects as well as malformation. AS is caused by the dysfunction of *UBE3A*, deletions (70 %), UPD (0–20 %) and aberrant methylation (2–5 %) in the maternal allele. AS presents with global developmental delay, convulsions, scoliosis, excessive laughter, movement and balance disorders, and sleep disturbance.

Both BWS and SRS are related to chromosome 11p15.5. The former is an overgrowth syndrome characterized by exomphalos, macroglossia, gigantism and an increased risk of developing embryonal tumors in childhood. It is a multigenic disorder resulting from genetic or epigenetic alterations of only the maternal allele. Hypermethylation on *H19* and hypomethylation on *LIT1* account for 50–60 % of sporadic patients. SRS is a clinically heterogeneous condition characterized by severe intrauterine growth retardation, poor postnatal growth, craniofacial features such as a triangular face and a broad forehead, body asymmetry, and a variety of minor malformations. Hypomethylation of *H19* at chromosome 11p15.5 (40 %) is known to be a frequent occurrence in SRS [30]. Various additional loci on chromosomes have been implicated as having a role in this syndrome [6, 24, 26, 27, 31]. Among these diseases, DNA methylation error (epimutation) rates are much higher for BWS and SRS patients, whereas the rates are much lower for PWS and AS. This might be expected since, while epimutations often account for BWS and SRS, they rarely do so for PWS and AS after ART. In fact, an increased frequency of AS patients after ART has been reported.

## Nationwide investigation of imprinting disorders

**Figure 3:**
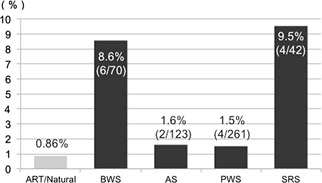
Association between imprint disorders and ART. ART/Natural: Children conceived with the use of ART comprised 0.86 % of the total number of births in 2009. Using this population rate, AS and PWS patients born after ART were found at similar frequencies to those from non‐ART births. However, using the same method, the numbers of BWS and SRS patients born after ART were nearly 10‐fold greater than the predicted frequencies

We performed a nationwide epidemiological study of the Japanese population to determine the frequency of four imprinting disorders, BWS, AS, PWS and SRS, during 2009 (Ministry of Health, Labor and Welfare of Japan: The Specified Disease Treatment Research Program). With the cooperation of a total of 1602 institutions (response rate: 56.3 %), 444 BWS patients, 949 AS patients, 2070 PWS patients and 326 SRS patients were identified. The frequencies of imprinting disorders after ART were 1.6 % (2/123) for AS, 1.5 % (4/261) for PWS, 8.6 % (6/70) for BWS and 9.5 % (4/42) for SRS, respectively. The contents of ART procedures were mostly IVF and ICSI: 81.2 % (13/16). Children born after ART were approximately 0.86 % of the total number of children born in Japan in 2009. Using this population rate of ART‐conceived babies, AS and PWS patients after ART were found at frequencies similar to those after natural conception. However, the numbers of BWS and SRS patients after ART were about 10–fold to 12‐fold greater than the predicted numbers because 50 % of the patients were not informative cases (Fig. 3) [32].

A limited number of studies have addressed the issue of childhood cancer, including retinoblastoma (RB), among children conceived after ART [33, 34]. In Japan, childhood cancer rates are also examined in patients with imprinting disorders. As expected, ~10 % of BWS patients developed several kinds of childhood cancer. Therefore, we need to be aware of the possibility of cancer development in childhood among such patients.

## Methylation patterns in imprinting disorders after ART

**Figure 4:**
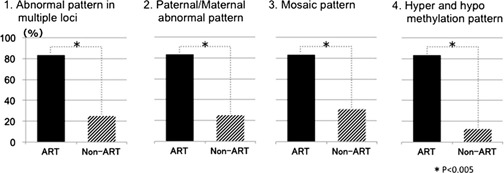
Comparison of abnormal methylation patterns in imprinting disorders after ART and natural conception (non‐ART). In most of the ART groups, DNA methylation was not restricted to one DMR and was present at both maternally and paternally methylated DMRs. Almost all cases showed a mixture of hypermethylation and hypomethylation. Furthermore, mosaic (incomplete) methylation patterns also were found. ART group: *n* = 6; Non‐ART group: *n* = 16. *P* < 0.005

These imprint‐associated disorders have been diagnosed by their characteristic clinical phenotypes, by FISH, by genetic and by epigenetic approaches. However, not enough analyses of DNA methylation errors (epimutations) are performed. It is known that 23 germline DMRs (gDMRs) are present in human chromosomes. The methylation status in some gDMRs within imprinted regions might be implicated in these syndromes. Detailed analysis of abnormal methylation patterns in imprinting disorders may provide clues as to the causes of disease and identify ART‐related risk factors. We analyzed 15 SRS samples (5 from after ART and 10 natural) with DNA methylation errors at *H19* DMR, and 7 BWS (1 ART and 6 natural) with DNA methylation errors of the *LIT1* DMR, and compared the DNA methylation status. In most of the ART samples, DNA methylation was not restricted to the *H19* DMR and was present at both maternally and paternally methylated gDMRs. Almost all cases showed a mixture of hypermethylation and hypomethylation. Furthermore, mosaic (incomplete) methylation patterns also were found. In contrast, only a few patients from natural conception showed similar DNA methylation errors at other loci (Fig. 4; Table 3) [32].

**Table 3:** Abnormal methylation in patients after ART with SRS and BWS

Case	ART	Abnormal imprint locus and methylation pattern				
SRS‐1	IVF‐ET	*H19* Hypo‐M (M)	*MEST* Hyper‐M	*PEG10* Hyper‐M (M)	*GRB10* Hyper‐M	*ZNF597* Hypo‐M
SRS‐2	IVF‐ET	*H19* Hypo‐M (M)				
SRS‐3	IVF‐ET	*H19* Hypo‐M (M)	*MEST* Hyper‐M (M)			
SRS‐4	IVF‐ET	*H19* Hypo‐M	*GRB10* Hyper‐M			
SRS‐5	IVF‐ET	*H19* Hypo‐M (M)	*INPP5F* Hyper‐M			
BWS‐1	ICSI	*LIT1* Hypo‐M	*ZDBF2* Hyper‐M	*MEST* Hyper‐M	*GNAS*‐*AS1* Hypo‐M (M)	

*Hypo‐M* Hypomethylation, *Hyper‐M* Hypermethylation (M): Mosaic (incomplete) methylation

The pattern of cellular mosaicism suggested that the imprinting defects occurred after fertilization rather than in the gamete, perhaps via a mechanism that impaired the maintenance of imprints. The mechanisms controlling the protection of imprinted loci against demethylation remain unclear, but the data suggest that this protection may fail in ART, resulting in tissue‐specific loss of imprints. Potential factors involved could include the culture conditions for the ovum and the length of exposure to specific media or growth factors as part of the ART procedure. Animal studies suggest that in vitro embryo culture may be associated with epigenetic alterations. In particular, the large offspring syndrome in cattle undergoing ART is associated with loss of maternal allelic methylation at *IGF2R* DMR [35] and has phenotypic similarity to BWS [36].

A comprehensive survey of all the known gDMRs in a number of patients with BWS and SRS revealed that multiple loci were more likely to be affected in the patients after ART than after natural conception. Lim et al. [37] reported a similar increased frequency of multiple errors after ART with 37.5 % of 25 patients after ART and 6.4 % of 55 naturally conceived patients displaying abnormal methylation at additional imprinted loci. However, Bliek et al. [38] demonstrated the alteration of multiple imprinted loci in 17 of 81 BWS patients with hypomethylation of *KCNQ1OT1*(*LIT1*) DMR; only one of this group with multiple alterations was born after ART. Similarly, Rossignol et al. [39] reported that 3 of 11 (27 %) patients born after ART and 7 of 29 (24 %) born after natural conception displayed abnormal methylation at additional loci other than the responsible locus. In these three studies, not all gDMRs were assayed, and it may be that by doing so, these incongruities will be resolved.

The increased frequency and difference of the patterns of DNA methylation errors between the two groups suggested that the BWS and SRS in the patients after ART might exhibit additional phenotypic characteristics. However, when the clinical features from both categories of conception were compared in detail, a significant difference was not found between ART and naturally conceived patients with BWS and SRS. The patients with diagnosed imprinting disorders having defects at additional loci other than the domain responsible for that disorder did not display additional phenotypes. It is, therefore, possible that the dysfunction of additional genes does modify the typical SRS and BWS phenotypes. Chang et al. [40] reported no phenotypic differences between ART and naturally conceived BWS patients. However, Lim et al. [37] reported that patients after ART had a significantly lower frequency of exomphalos and a higher risk of non‐Wilms’ tumor neoplasia. Phenotypic differences between ART and naturally conceived patients are largely unreported, and any changes of phenotype may be altered by the frequency and the degree of epimutations. Studies revealed that patients with BWS born after ART presented with epimutations that were not restricted to the 11p15 region [37, 38, 39]. There is a recently recognized BWS‐like syndrome involving overgrowth with severe developmental delay reported after IVF/ICSI [41]. Further analysis of abnormal methylation patterns in imprinting disorders may provide clues as to the causes of disease and identify the ART‐related risk factor(s).

## Effect of ART on human gametes and embryos

1. *Ovulation induction*. For humans, studies on imprinting reprogramming during oogenesis are very limited due to material collection and ethical reasons. Proper control oocytes are scarce and are confounded by maternal age and/or general suboptimal oogenesis. In a study of MI (metaphase) and GV (germinal vesicle) oocytes, around 60–70 % were methylated at *KCNQ1OT1* DMR, whereas in MII oocytes, which are used for IVF/ICSI treatment, the methylation level was 90, and 10 % were found to have aberrant methylation [42]. Regarding the expected paternal *H19* DMR demethylation in oocytes, some MI/GV oocytes were reported to have methylated alleles after ovarian stimulation [24].
2. *In vitro maturation*. IVM of oocytes has been introduced to retrieve oocytes for IVF treatment avoiding exogenous gonadotrophins, especially for patients at risk for ovarian hyperstimulation syndrome and/or polycystic ovary syndrome [43]. Immature oocytes at the antral follicle stage are cultured for 24–48 h before fertilization. Khoueiry et al. [42] reported that the methylation level of *KCNQ1OT1* DMR was significantly lower in IVM‐derived MII oocytes and pointed out that the maturation time might be too short to finish the methylation process.
3. *Male subinfertility*. Several studies show that disturbed spermatogenesis itself is associated with incorrect imprinting. In spermatozoa from oligozoospermic men, the occurrence of hypermethylation of several maternally imprinted DMRs or hypomethylation of paternally DMRs is increased [44, 45, 46, 47, 48]. Boissonnas et al. [49] reported the association between methylation and the sperm concentration in teratozoospermic (TZ) and oligo‐astheno‐teratozoospermic (OAT) patients. In spermatozoa from TZ patients, only 2 of 16 CpG sites at *H19* DMR (CTCF6 region) were hypomethylated. In OAT spermatozoa, methylation was drastically reduced for all CpGs. OAT spermatozoa also show reduction in another paternal DMR, IG‐DMR methylation [50]. Alteration of the protamine 1 to protamine 2 ratio generally denotes affected spermatogenesis and leads to hypermethylation of several maternally imprinted DMRs and hypomethylation of paternal DMRs [48]. Azoospermia caused by anejaculation and secondary inflammatory obstruction is related to an increase in maternal DMRs [51]. The methylation of non‐imprinted genes and a repetitive sequence were also affected [52], typically for sequences showing large intraindividual and interindividual methylation variations in spermatozoa from normozoospermic males [53].
4. *Effect on IVF outcome*. It is not known to what extent the degree and prevalence of DMR CpG methylation can be ablated before germline transmission of this mark suffers. Kobayashi et al. [46] found that abnormal methylation in trophoblastic villi from ART‐miscarriages was transmitted with the abnormal imprints in semen from the father. In a patient with hypospermatogenesis and almost complete hypomethylation of the *H19* DMR, the embryos obtained after ICSI all showed developmental arrest [51]. There is a case report in which part of a methylation defect (SRS) of a child conceived by IVF was also detected in leukocytes from the father [54].
5. *Effect of embryo culture*. Among low‐quality human surplus embryos not suitable for transfer and cryopreservation, 19 % showed hypomethylation of *H19* DMR [55]. Similar results were obtained in another study that examined the methylation of the corresponding sperm samples and found a normal pattern [56]. It is not known whether hypomethylation leads to growth arrest or whether the growth arrest leads to loss of methylation. Recently, Dumoulin et al. [57] reported that IVF culture of embryos in two different media resulted in a significant difference in birth weight of almost 250 g.
6. *Epigenetic effects of IVF on offspring*. Except for the described imprinting disorders, induced epigenetic variations that do not have clear phenotypical effects might be transmitted to offspring. In chorion villus samples from spontaneous miscarriages and stillbirth, Zechner et al. [58] demonstrated hypomethylation of *KCNQ1OT1* as well as *H19* in samples derived after IVF. The intraindividual and interindividual variations in methylation are higher in placental tissue than in umbilical cord blood but also increase after IVF compared with natural fertilization [59]. Extended DNA methylation analysis including DMRs in placental tissue and umbilical cord blood from IVF and control pregnancies indicated that imprinted genes were not more vulnerable to methylation differences than non‐imprinted genes [60]. Approximately 15 % of CpG sites showed a difference in methylation in placental tissue, as did 20 % in umbilical cord blood.
7. *Physiological outcomes of children born after IVF*. Ceelen et al. [61] analyzed the physical development of children born after IVF. They compared blood pressure, skinfold thickness, fasting glucose/insulin levels, fat, growth velocity, bone development and endocrine status during puberty. These were all higher in the IVF group than in a control group. Sakka et al. [62] and Miles et al. [63] also reported similar results. However, these studies found no genetic component indicating that children conceived via ART were different from those conceived naturally.

## Conclusions

**Figure 5:**
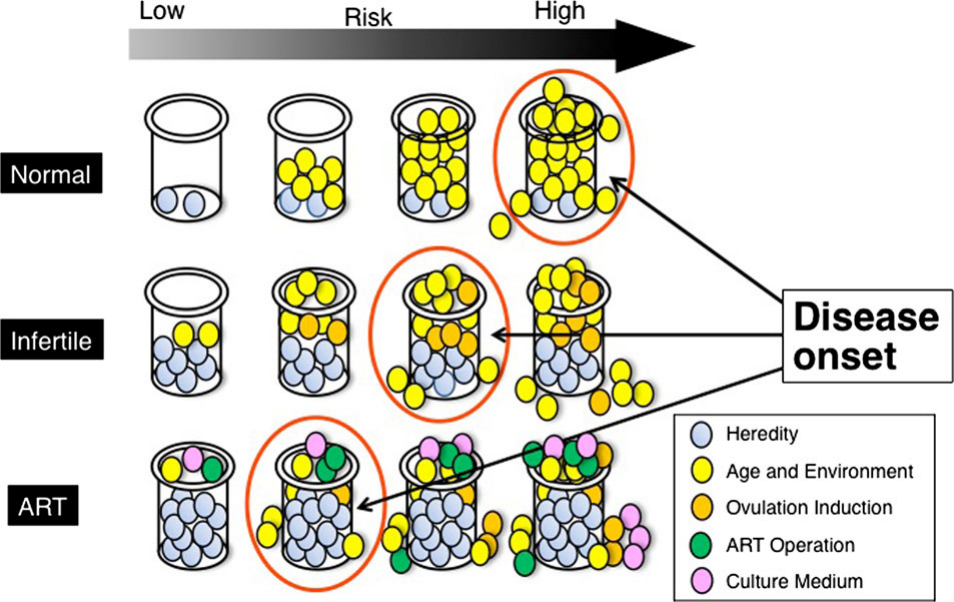
Model of the onset of imprinting disorders after ART. Our model of onset of imprint‐associated disorders shows that a combination of factors such as the process of ART, infertility and advanced maternal age are likely to account for the increases in the diseases as synergy effects

It is still unknown when imprinting errors arise and what factors predispose to epigenetic changes. Both IVF and ICSI appear to be associated with an increased relative risk of imprinting disorders [64]. The process of ART, which includes hormone stimulation, in vitro culturing, and cryopreservation, and the timing of embryo transfer have been shown to influence the proper establishment and maintenance of genomic imprints in the developing epigenome. Some infertile males, particularly those with oligozoospermia, carry preexisting imprinting errors in their sperm. Therefore, both the process of ART and infertility might contribute to the risk of imprinting disorders. Advanced maternal child bearing age is a risk factor for the development of PWS, which is caused via non‐junction at meiosis I [65, 66, 67]. We, therefore, made a model including a combination of various factors (Fig. 5).

The key finding from these studies was a clear association between ART and specific imprinting disorders. In addition, the association between ART and a more global disruption of genomic imprints was demonstrated. The increased frequency of imprinting disorders after ART is perhaps not surprising given the major epigenetic events that take place during early development at a time when the epigenome is most vulnerable. What is particularly intriguing is why some disorders such as BWS, SRS and AS are more associated with ART than others such as PWS. This could suggest that some loci are more responsive to external events.

There is a pressing need to examine a larger number of imprinting disorders and conduct a long‐term international follow‐up study of the results of ART treatment, particularly as the use of ART increases worldwide. These rare disorders are on the increase, and it is not yet known what other pathologies may be influenced by ART. For example, in addition to general growth abnormalities, many imprint methylation errors also lead to the occurrence of various cancers and mental diseases [68, 69]. Further molecular studies are required to understand the pathogenesis of this association and what precautions can be taken to prevent the occurrence of these syndromes. We hope that the constitution of children born after each ART procedure will reveal the safest and most ethical approach to use, which will be invaluable for the future development of standard ART treatments.

## Acknowledgments

The authors thank the patients and their families who participated in this study. We are also grateful to the physicians who responded to the surveys. We would like to thank all the members of our laboratory for their technical assistance, support and valuable suggestions.

### Conflict of interest

The authors declare that they have no conflict of interest.
